# Transcriptome sequencing and whole genome expression profiling of hexaploid sweetpotato under salt stress

**DOI:** 10.1186/s12864-020-6524-1

**Published:** 2020-03-04

**Authors:** Mohamed Hamed Arisha, Hesham Aboelnasr, Muhammad Qadir Ahmad, Yaju Liu, Wei Tang, Runfei Gao, Hui Yan, Meng Kou, Xin Wang, Yungang Zhang, Qiang Li

**Affiliations:** 1 Xuzhou Institute of Agricultural Sciences in Jiangsu Xuhuai District / Key Laboratory of Biology and Genetic Improvement of Sweetpotato, Ministry of Agriculture / Sweetpotato Research Institute, CAAS, Xuzhou, 221131 Jiangsu China; 2 Department of Horticulture, Faculty of Agriculture, Zagazig University, Zagazig, Sharkia 44511 Egypt; 3 Plant pathology department, Agriculture and Biology research division, National research center, Giza, Egypt; 4 Department of Plant Breeding and Genetics, Bahauddin Zakariya University, Multan, 60000 Pakistan

**Keywords:** Hexaploid sweetpotato, Salt stress, Expression profile, RNA-sequencing, Transcriptome

## Abstract

**Background:**

Purple-fleshed sweetpotato (PFSP) is one of the most important crops in the word which helps to bridge the food gap and contribute to solve the malnutrition problem especially in developing countries. Salt stress is seriously limiting its production and distribution. Due to lacking of reference genome, transcriptome sequencing is offering a rapid approach for crop improvement with promising agronomic traits and stress adaptability.

**Results:**

Five cDNA libraries were prepared from the third true leaf of hexaploid sweetpotato at seedlings stage (Xuzi-8 cultivar) treated with 200 mM NaCl for 0, 1, 6, 12, 48 h. Using second and third generation technology, Illumina sequencing generated 170,344,392 clean high-quality long reads that were assembled into 15,998 unigenes with an average length 2178 base pair and 96.55% of these unigenes were functionally annotated in the NR protein database. A number of 537 unigenes failed to hit any homologs which may be considered as novel genes. The current results indicated that sweetpotato plants behavior during the first hour of salt stress was different than the other three time points. Furthermore, expression profiling analysis identified 4, 479, 281, 508 significantly expressed unigenes in salt stress treated samples at the different time points including 1, 6, 12, 48 h, respectively as compared to control. In addition, there were 4, 1202, 764 and 2195 transcription factors differentially regulated DEGs by salt stress at different time points including 1, 6, 12, 48 h of salt stress. Validation experiment was done using 6 randomly selected unigenes and the results was in agree with the DEG results. Protein kinases include many genes which were found to play a vital role in phosphorylation process and act as a signal transductor/ receptor proteins in membranes. These findings suggest that salt stress tolerance in hexaploid sweetpotato plants may be mainly affected by TFs, PKs, Protein Detox and hormones related genes which contribute to enhance salt tolerance.

**Conclusion:**

These transcriptome sequencing data of hexaploid sweetpotato under salt stress conditions can provide a valuable resource for sweetpotato breeding research and focus on novel insights into hexaploid sweetpotato responses to salt stress. In addition, it offers new candidate genes or markers that can be used as a guide to the future studies attempting to breed salt tolerance sweetpotato cultivars.

## Background

Sweetpotato (*Ipomoea batatas* (L.) Lam.), the only crop plant belongs to *Convolvulaceae* family with starchy storage roots. Purple-fleshed sweetpotato (PFSP) considered to be an important source for anthocyanin which displays strong antioxidant properties [1]. It is also considered as an important staple source of calories and proteins which consumed by all age groups. In terms of agricultural production sweetpotato considered as the seventh most important food crop in the world [2].

Salinity is a global problem caused vast area of lands remaining uncultivated. Exposure of sweetpotato plants to salt stress resulting in problems such as ion imbalance, mineral deficiency, osmotic stress, ion toxicity and oxidative stress [3]. Ultimately, these conditions interact with several cellular components including DNA, protein, lipids and pigments. That’s in rule impeding plant development and affect sweetpotato production [4]. Therefore, introducing of salt tolerant sweetpotato cultivar became necessary.

With the fact of environmental stress and climate change there is an urgent need to accelerate crops breeding with higher production and stress tolerance traits [5]. In sweetpotato transcriptome sequencing offers a rapid approach for crop improvement with promising agronomic traits and stress adaptability. Several transcriptome sequencing studies have been conducted on hexaploid sweetpotato genome [6–8]. However, having a complex genome structures (2n = 6x = 90), sweetpotato still didn’t achieve a reference genome which covered a few percent of genome, so still a long way from the reference genome [9].

Currently, referring to the potential advantages of anthocyanin for health, more attention was paid to transcriptome analysis of purple flesh sweetpotato [10]. Most of conducted transcriptome sequencing on PFSP focused on genes related to anthocyanins biosynthesis and their regulation mechanism [11, 12]. While, few researches have been done on the effect of biotic or abiotic stress on PFSP.

In the present study, second and third generation sequencing technology were used to establish a useful database of transcriptomes sequencing as well as differentially expressed genes in sweetpotato leaves under salt stress conditions. In total 102,845,433 high quality reads were assembled into 16,856 transcripts giving 15,998 unigenes. Our results provide novel insights into hexaploid sweetpotato response to salt stress and identified numerous specific genes involved in salt stress defense mechanisms. That’s in role can be used to guide future efforts towards breeding of sweetpotato salt resistant cultivars.

## Results

### Sequencing and de novo assembly of sweetpotato transcriptome under salt stress conditions

**Fig. 1:**
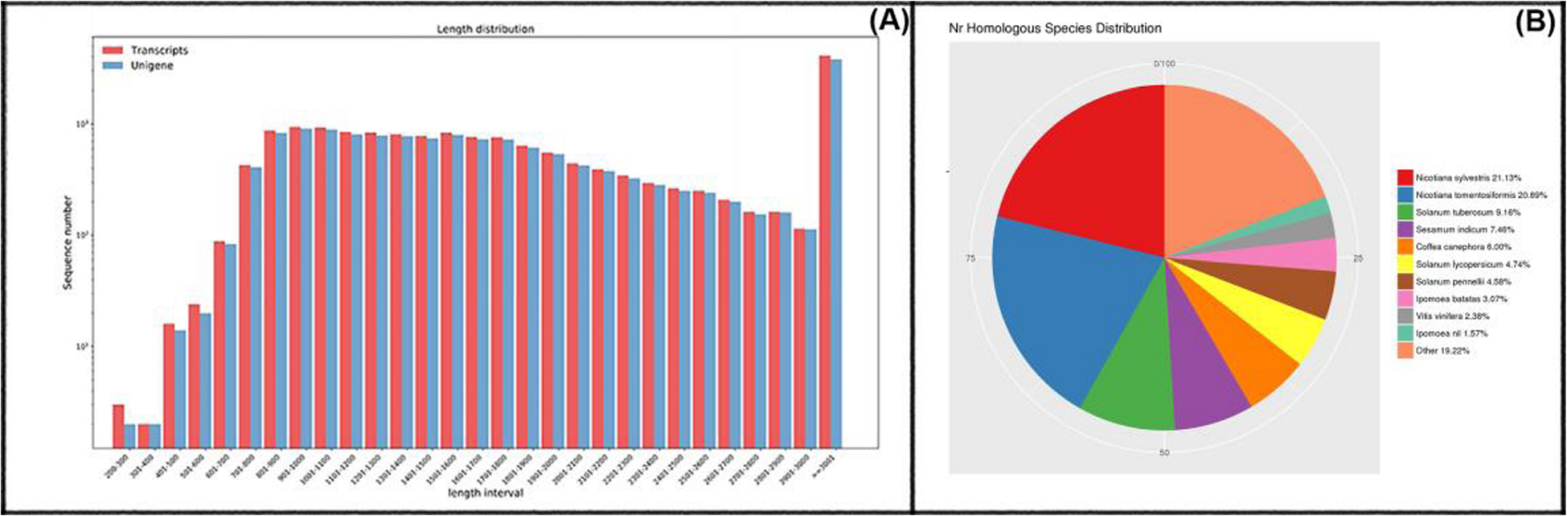
**a** Assembly result sequence length distribution map of transcripts and unigenes in Xuzi-8 sweetpotato cultivar. The horizontal axis represents the length intervals of the transcripts and unigenes, and the vertical axis represents the number of transcripts and unigenes. **b** Species distribution of the top BlastX matches of the transcriptome unigenes of Xuzi-8 sweetpotato cultivar in the non-redundant protein database (Nr) data base

For NGS, five cDNA libraries were prepared from the third true leaf of PFSP seedlings (Xuzi-8 cultivar) treated with 200 mM NaCl for 0, 1, 6, 12, 48 h. These libraries were separately sequenced using Illumina high-throughput second generation sequencing platform. After removing the low-quality reads and all possible contaminations, a total of 170,344,392 clean reads with Q20 > 96.73% and GC percentage between 45.07 and 46.50% were used for further study (Table 1). Each library was represented by over than 30 million high-quality reads, with number ranging from 32,830,183 to 35,663,873. For 3rd GS, four time points RNA samples including1, 6, 12 and 48 h were mixed to produce one library beside to the control library. These libraries were separately sequenced using Illumina high-throughput third generation sequencing platform. TPM, FPKM, RPKM and fold change (FC) were recorded for each replicate of each library separately on both NGS and 3rd GS. Obtained sequence from NGS and 3rd GS were aligned and similar sequence data from all libraries/samples were pooled. Due to the lack of a reference genome, the clean reads resulted in from the transcriptome sequences were aligned and assembled using Trinity software. After further clustering and assembly, a total of 21,497,466; 20,272,643; 21,954,725; 19,121,890 and 19,998,709 mapped reads were obtained with percentage 60.26, 61.79, 61.62, 59.33 and 58.87% of total reads at different time points (0, 1, 6, 12, 48 h), respectively. As shown in Table 1 that the average length of transcripts and unigenes was more than 2000 bp which indicate that the obtained data are high quality data**.** Statistics on unigenes and transcripts length resulted from mixed second and third generations sequencing were performed using PacBio’s officially recommended cogent software (Tables 1 and 2, Fig. 1). In addition, the total number of CDS was 30,615 of which 23,245 CDS mapped to the protein database.

**Table 1:** Next generation sequencing statistical summary of sequenced and assembled results

	0 h	1 h	6 h	12 h	48 h
Total Reads	35,663,873	32,830,183	35,632,937	32,241,116	33,976,283
Mapped Reads	21,497,466	20,272,643	21,954,725	19,121,890	19,998,709
Mapped Ratio	60.26%	61.79%	61.62%	59.33%	58.87%
Nt	10,699,162,000	9,849,054,900	10,689,881,200	9,672,334,900	10,192,884,900
GC (%)	45.53	46.50	46.17	45.29	45.07
Q20 (%)	96.72	96.63	96.77	96.75	96.77
Q30 (%)	92.03	91.66	92.08	91.91	91.96
N Percentage	00.00	00.00	00.00	00.00	00.00

Note: Nt, total number of clean nucleotides; The GC percentage is the proportion of guanidine and cytosine nucleotides among total nucleotides; The Q20 and Q30 percentage is the proportion of nucleotides with a quality value >20 and 30, respectively; The N percentage is the proportion of unknown nucleotides in clean reads

**Table 2:** Third generation sequencing statistical summary of sequenced and assembled results

	Unigenes	Transcripts
Total number of sequences	15,998	16,856
Total sequences length	34,848,832	36,928,928
Maximum length	9,135	9,135
Minimum length	208	208
Average length	2,178	2,190
Percent GC	43.10%	43.12%
N40	3,698	3,733
N50	2,652	2,701
N60	2,131	2,155
N70	1,785	1,795
N80	1,503	1,509
N90	1,162	1,168

N50, represents sorting the assembled transcripts from long to short by length, accumulating the length of the transcript to 50% of the total length, corresponding to the length of the transcript, and so on

### Functional annotation

To annotate the obtained unigenes, a BlastX search against the NR NCBI protein database with cut-off E-value of 10^− 5^ based on sequence similarity was performed. In total, 15,461 unigenes were detected (Table 3) that showed comparability with known gene sequence in all databases corresponding to approximately 96.64% of total unigenes including Clusters of orthologous groups (COG), Gene ontology (GO), Kyoto encyclopaedia of genes and genomes (KEGG), eukaryotic orthologous group (KOG), protein family (PFAM), Swiss-Prot., NCBI non-redundant protein sequences (Nr). According to Fig. 1b, the species that gave the best BlastX matches were *Nicotiana sylvestris* (21.30%) followed by *Nicotiana tomentosiformis* (20.69%), *Solanum tuberosum* (9.16%), *Sesamum indicum* (7.46%), *Coffea canephora* (6.00%), *Solanum lycopersicum* (4.74%), *Solanum penellii* (4.58%), *Ipomoea batatas* (3.07%), *Vitis vinifera* (2%), *Ipomoea nil* (1.57%) and others (19.22%).

**Table 3:** Statistics of unigenes annotated in public database

Annotated Database	Annotated Number Value (%)	300<=length<1000 Value (%)	length>=1000 Value (%)
COG	6982 (43.64%)	853 (12.22%)	6129 (87.78%)
GO	12480 (78.01%)	1750 (14.02%)	10728 (85.96%)
KEGG	5965 (37.29%)	806 (13.51%)	5159 (86.49%)
KOG	10020 (62.63%)	1164 (11.62%)	8856 (88.38%)
Pfam	13569 (84.82%)	1725 (12.71%)	11844 (87.29%)
Swiss-Prot.	13428 (83.94%)	1675 (12.47%)	11751 (87.51%)
Nr	15446 (96.55%)	2115 (13.69%)	13329 (86.29%)
All	15461 (96.64%)	2125 (13.74%)	13334 (86.24%)

### Gene ontology (GO) and KOG classifications

**Fig. 2:**
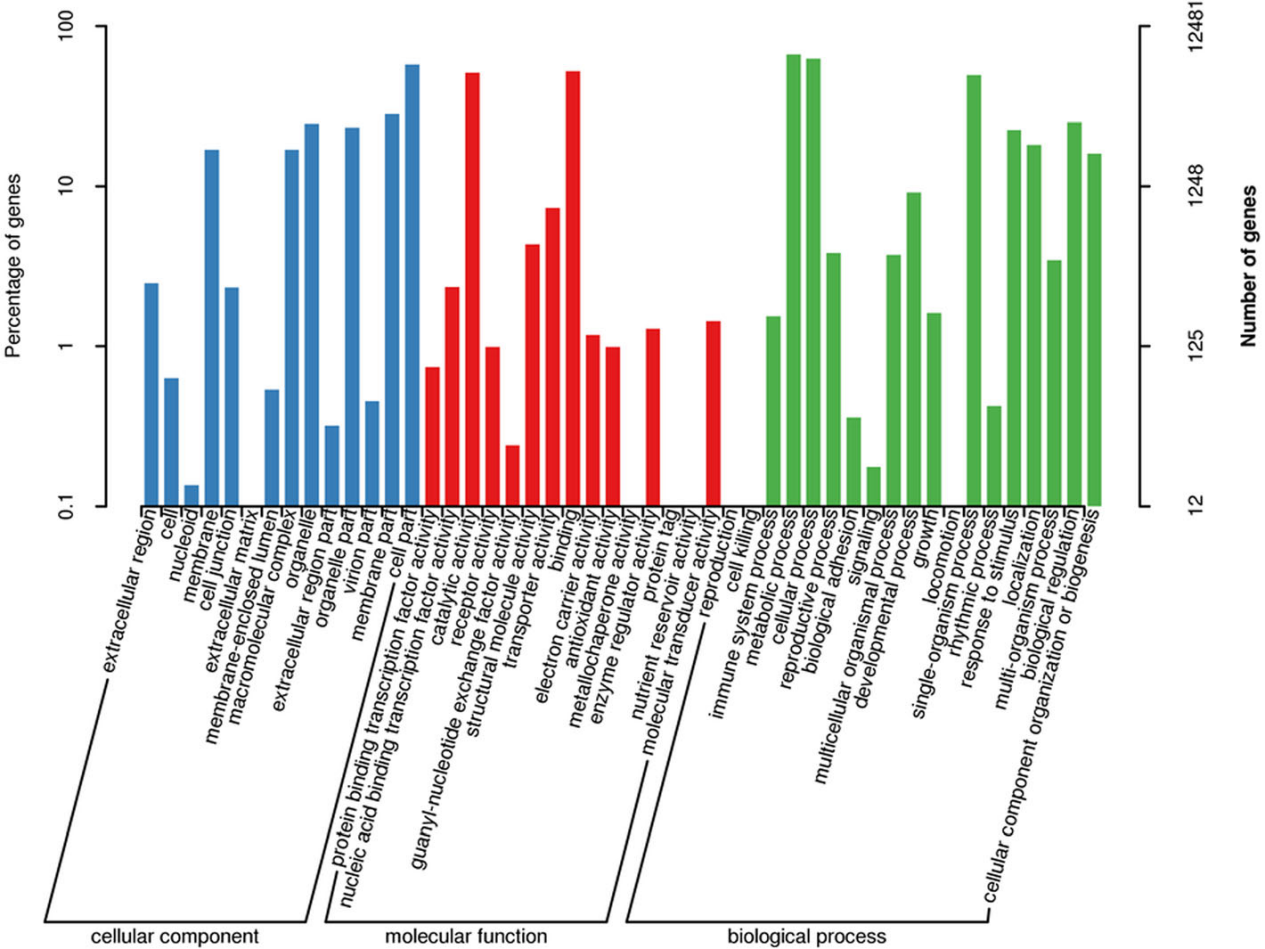
Gene ontology (GO) classifications in sweetpotato (Xuzi-8 cultivar), the percentage indicate the proportion of unigenes with the GO annotations

For functional categories of 15,998 successfully annotated unigenes, a total of 12,481 genes (78.01%) (Table 3 and Additional file 1) were assigned to at least one GO term. These GO terms were categorized into 48 functional groups which were divided into three categories including biological process, cellular component and molecular function (Fig. 2 and Additional file 2). For biological process, the highest categories were metabolic (8291 unigenes, 53.55%) followed by cellular process (7774, 50.21%) then single organism process (6181, 39.92%). In the category of molecular function, the most abundant groups included catalytic activity (6369, 41.14%) and binding activity (6513, 42.07%). Furthermore, the most abundant group for cellular components was cell parts (7198, 46.49%) (Additional file 3).

**Fig. 3:**
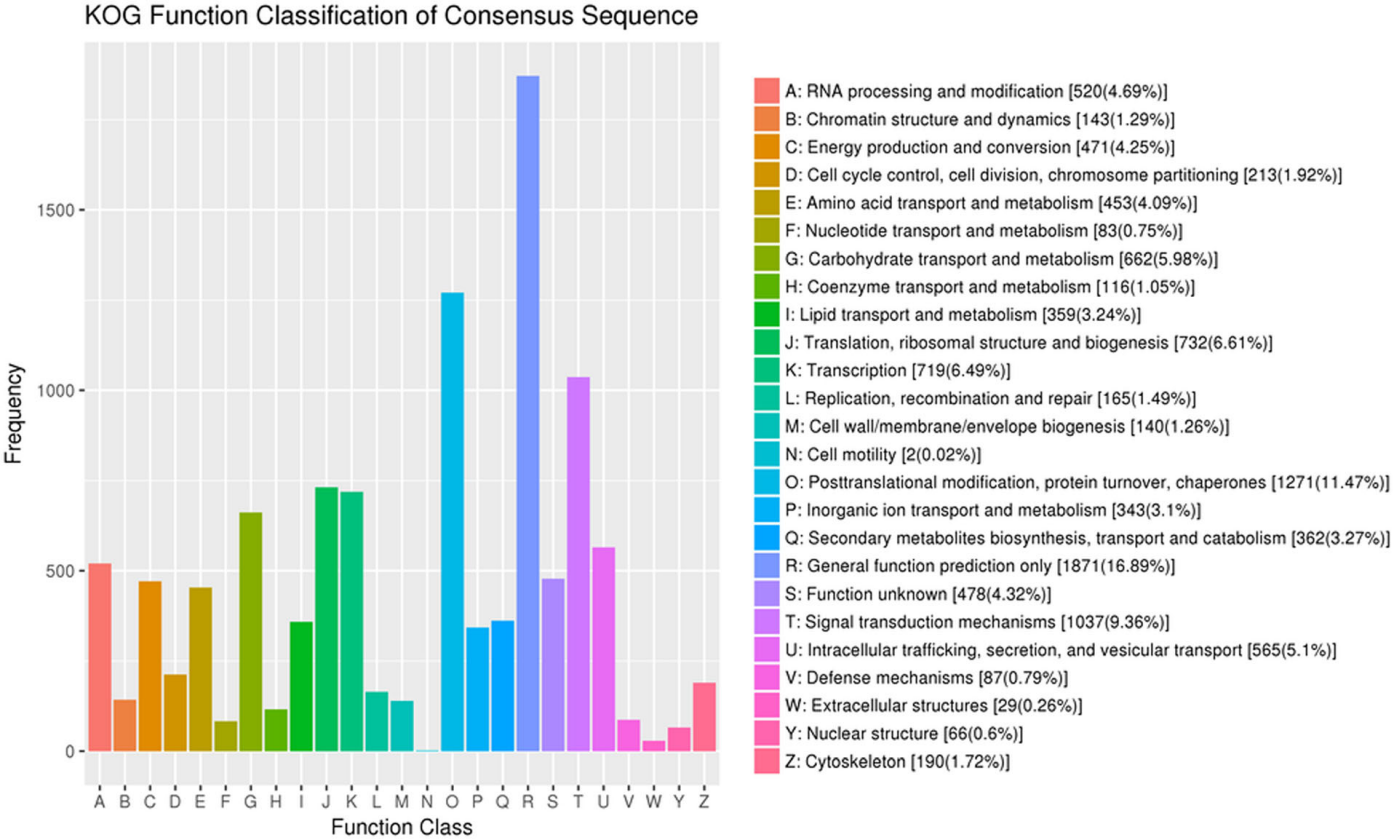
Clusters of orthologous groups (COG) classification in Xuzi-8 sweetpotato cultivar. Genes from the same Orthologous have the same function, so that direct functional annotations to other members of the same KOG cluster

Genetic orthologous relationships, combines evolutionary relationships were used to classify the potential functions into different orthologous clusters (COG). In total 10,020 genes were subdivided into 25 functional classes as shown in Fig. 3 and Additional file 1. Among the 25 groups, “general function prediction only” represented the largest group (1871 unigenes, 16.89%) followed by “post translational modification, protein turnover, chaperons” (1271 unigenes, 11.47%) then “signal transduction mechanisms” (1037 unigenes, 9.36%). In addition, it was interesting to note that 87 genes were aligned to the “defense mechanisms” cluster (Fig. 3). Rigorous algorithm (FDR ≤ 0.001, log2 FC-ratio ≥ 1) were applied to measure the significance level of the 87 obtained genes. Out of these defense mechanisms genes there were no significant expressed genes during the first hour of salt stress. Furthermore, there were two unigenes, (unigene802 and 1120) which significantly up-regulated during 6, 12 and 48 h of salt stress, these two unigenes were aligned to GID1-like gibberellin receptor. On the other hand the unigene6088 (cinnamoyl-Co-A reductase 1- like) was significantly down-regulated at 6 h of salt stress. In addition, at 48 h of salt stress, there were two unigenes (5647 and 5851) significantly down-regulated which were aligned to Alpha/beta hydrolase (carboxylesterase) and cinnamoyl-CoA reductase 1-like (Additional file 1).

### KEGG annotations

**Fig. 4:**
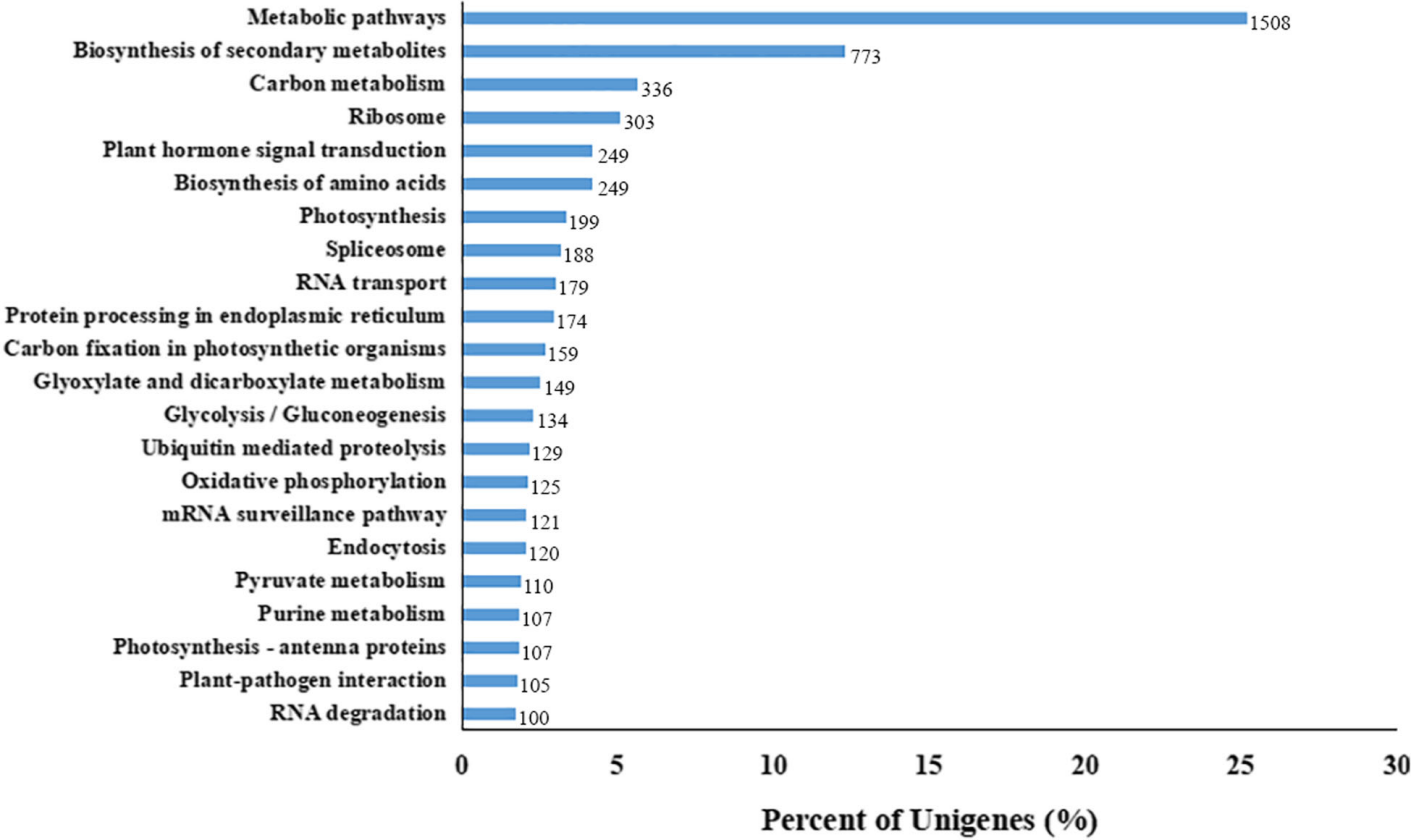
The most enriched KEGG clusters in Xuzi-8 sweetpotato cultivar. The most enriched 22 clusters out of 123 clusters were presented in this figure

KEGG pathway annotation for 15,461 unigenes was obtained as shown in Fig. 4. A total of 5965 sequences were assigned to 125 pathways. The largest enriched groups in the KEGG pathways were “Metabolic pathways (ko01100)” (1508 unigenes, 25.28%) and “Biosynthesis of secondary metabolites (ko01110)” (733 unigenes, 12.28%), which ranked at 1st position. Followed by “Carbon metabolism (ko01200)” (336 unigenes, 5.63%), “Ribosome (ko03010)” (303 unigenes, 5.08%), “Plant hormone signal transduction (ko04075)” (249 unigenes, 4.17%), “Biosynthesis of amino acids (ko01230)” (249 unigenes, 4.17%) and “Photosynthesis (ko00195)” (199 unigenes, 3.34%). These specific enrichments KEGG pathways and mechanisms are involved in response to salt stress in sweetpotato (Xuzi-8 cultivar) (Additional file 4).

### Expression patterns of hexaploid sweetpotato unigenes in response to salt stress

**Fig. 5:**
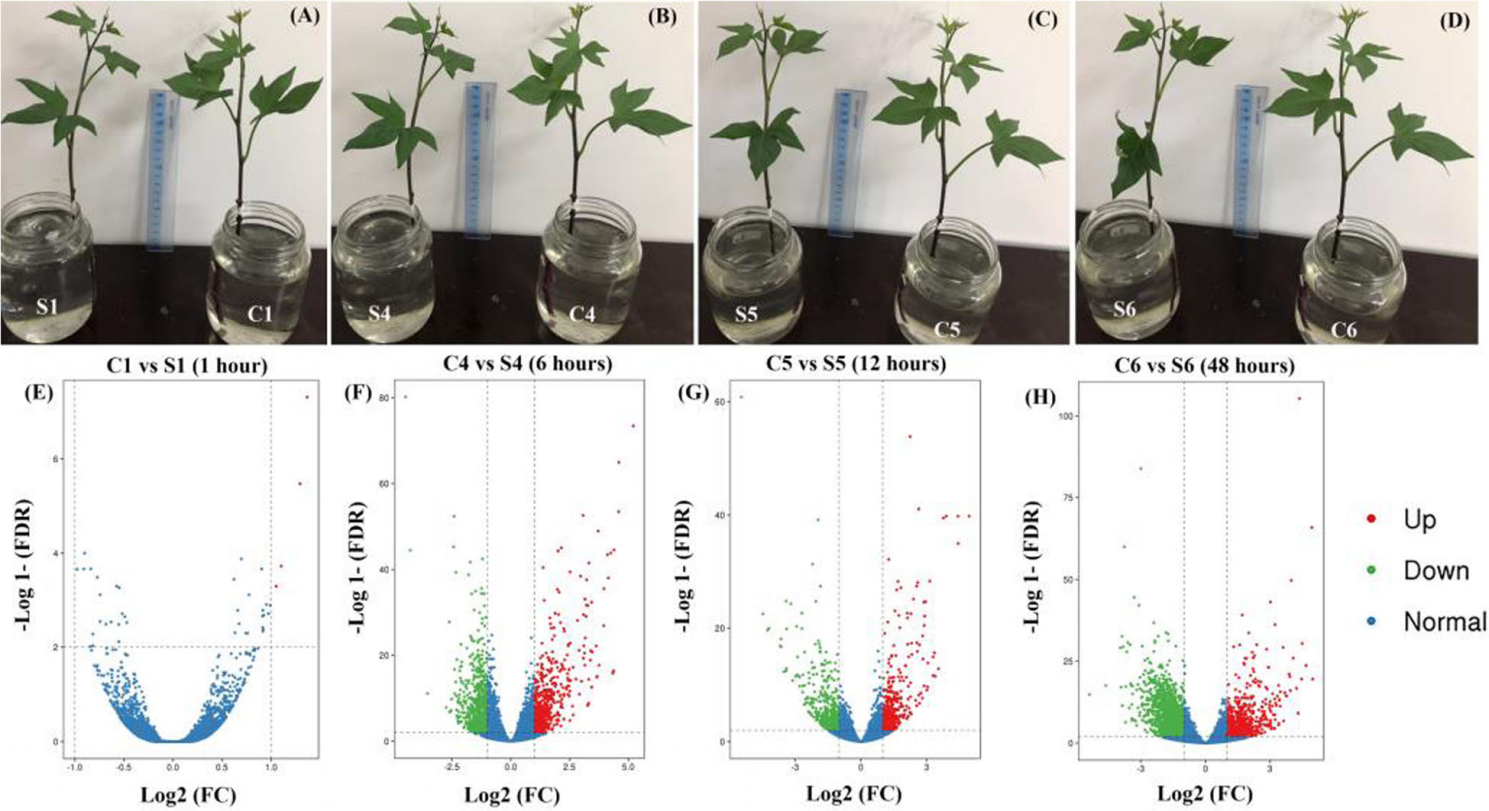
Phenotypic variations in Xuzi-8 sweetpotato seedlings as related to fold change (FC) and false discovery rate (FDR) under salt stress (200 Mm NaCL). **a**, **b**, **c** and **d**; phenotypic variations at 0, 1, 6, 12 48 hours of salt stress, respectively. **e**, **f**, **g** and **h**; fold change (FC) and false discovery rate (FDR) at four libraries 1, 6, 12 48 hours, respectively as compared to control

The results in Fig. 5 (a-d) showed the phenotypic changes during salt stress exposure as compared to control. Salt stress visual symptoms in the form of welting started slightly at 12 h and increased gradually showing slight leaves folding at 48 h. The highest number of DEGs was induced at 48 h of salt stress followed by 6 h and 12 h, respectively, while 1 h gave the lowest number of DEGs. Transcriptional level at 1, 6, 12, 48 h as compared to control induced expression values 4, 529, 341 and 663 as up-regulated unigenes, and 0, 672, 422 and 1531 as down-regulated, respectively. Furthermore, there were 15,534; 14,450; 14,703 and 13,330 normally expressed unigenes during 1, 6, 12 and 48 h of salt stress. In addition, there were 119 up-regulated genes, 87 down-regulated genes, 12,384 genes normal and 211 unknown genes common under all durations of salt stress (Fig. 5 e-h).

### Detection of salt-induced genes related to salt tolerance

RPKM read counts were used to identify DEGs significance level between control and salt-stressed samples using the rigorous algorithm (FDR ≤ 0.001, log2 FC-ratio ≥ 1) for significantly up-regulated unigenes and (FDR ≤ 0.001, log2 FC-ratio ≤ − 1) for significantly down regulated genes. Furthermore, number of 4, 479, 281, 508 unigenes were up-regulated with significant expression level in salt stress treated samples at the different time points of salt stress including 1, 6, 12, 48 h, respectively. On the other hand, there were 567, 301, 1335 unigenes significantly down-regulated at 6, 12, 48 h of salt stress (Fig. 7).

**Fig. 6:**
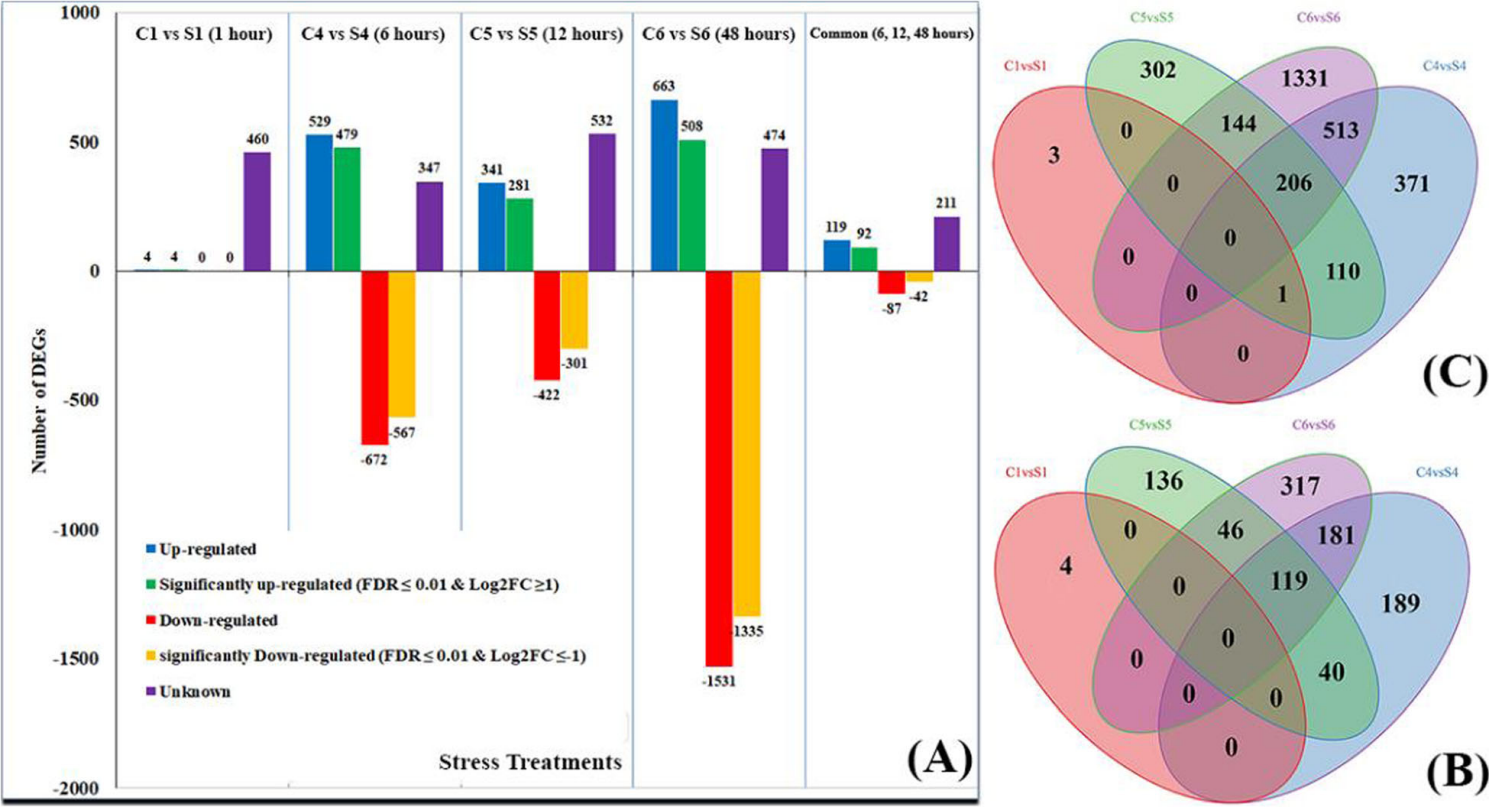
Comparison of four transcriptomes for classification of DEGs and statistics of sequence annotation of DEGs. **a**; Statistical chart of DEGs transcriptome in response to salt stress. Transcriptional level of five libraries including 1, 6, 12 and 48 hours of salt stress treatment as compared to control. **b** and **c**; Venn diagram analysis of up-regulated unigenes and all induced unigenes, respectively.

During the first hour of salt stress there were four significantly expressed unigenes including SBP-domain, HSP-70, pectin methyl esterase inhibitor, and uncharacterized protein sequence gene families, respectively (Fig. 6 and Table 4).

**Table 4:** Salt stress defence mechanism related genes, Genes involved in physiological processes related to response to salt stress and highest significantly expressed genes that were significantly up and down regulated under different durations (1, 6, 12, 48 hours) of salt stress (200 mM NaCl) as compared to control

Library	Unigene ID	Fold change	Nr. annotation	Unigene ID	Fold change	Nr. annotation
	Up-regulated			Down-regulated		
C1 vs S1	g11357	1.1	SBP-domain	-	-	-
	g15171	1.3	Hsp70			
	g16391	1.1	plant invertase/ pectin (methyl-esterase inhibitor)			
	g7424	1.4	Uncharacterized protein family			
C4 vs S4	Salt stress defence mechanism related genes					
	g1120		GID1- like gibberellin receptor	g6088	1.1	Cinnamoyl-Co-A reductase-1 like
	g802		GID1- like gibberellin receptor			
	Genes involved in physiological processes related to response to salt stress					
	g243	1.8	ABA-insensitive like protein (bZIP-TF)	g1047	1.1	Plastid glutamine synthase
	g3644	1.9	ABA-insensitive like protein (bZIP-TF)	g10527	2.0	Cellulose synthase A-catalytic [UDP-forming]
	g7343	3.6	EID-like F-box protein-3	g11093	1.9	Cellulose synthase A-catalytic [UDP-forming]
	g7826	1.6	WCOR-413-like cold accumulation protein	g11199	2.0	Cellulose synthase A-catalytic [UDP-forming]
	g9718	2.1	Putative low temperature and salt responsive protein isoform-1	g11449	1.9	Cellulose synthase A-catalytic [UDP-forming]
				g146	1.4	Dicarboxylate transporter-1, chloroplast
				g14716	2.1	Indole-3 acetic acid amino synthase [GH3]
				g15096	1.1	Indole-3 acetic acid amino synthase [GH3]
				g15564	1.1	Indole-3 acetic acid amino synthase [GH3]
				g1634	1.3	Tubulin alpha-2 chain-like
				g16418	1.6	Dicarboxylate transporter-1, chloroplast
				g16788	1.4	Dicarboxylate transporter-1, chloroplast
				g1835	1.1	Protein WALLS ARE THIN-1 like
				g6333	2.3	Aquaporin protein-12
				g6414	2.0	Aquaporin protein-12
	Highest significantly expressed genes (more than four folds higher than control)					
	g128	4.4	Protein TRANSPARENT TESTA-12 (MaTE protein detoxification)	g12981	4.5	Nitrate reductase [NADH]
	g16087	4.6	Malate synthase, glyoxysomal [PRUPE]	g13113	4.5	Nitrate reductase [NADH]
	g16131	4.3	Malate synthase, glyoxysomal [PRUPE			
	g2278	5.2	Malate synthase, glyoxysomal [PRUPE]			
	g4002	4.3	SNF-1 related protein kinase			
	g55	4.4	Malate synthase, glyoxysomal [PRUPE]			
	g5655	4.6	Malate synthase, glyoxysomal [PRUPE]			
	g9231	4.2	Dehydrin			
	g9447	4.1	Dehydrin			
C5 vs S5	Salt stress defence mechanism related genes					
	g1120		GID1- like gibberellin receptor	-	-	-
	g802		GID1- like gibberellin receptor			
	Genes involved in physiological processes related to response to salt stress					
	g13368	1.2	BEL-like homes domain protein-1 (BLH-1)	g10527	1.8	Cellulose synthase A-catalytic [UDP-forming]
	g13566	1.0	BEL-like homes domain protein-1 (BLH-1)	g11199	2.0	Cellulose synthase A-catalytic [UDP-forming]
	g243	2.9	ABA-insinsative like protein (bZIP1-TF)	g11449	2.0	Cellulose synthase A-catalytic [UDP-forming]
	g3644	3.3	ABA-insinsative like protein (bZIP1-TF)	g1634	1.3	Tubulin alpha-2 chain-like
	g7343	3.9	EID-like F-box protein-3	g2681	1.1	Enoyl-[acyl-carrier-rotein] reductase [NADH], chloroplast-like
				g11093	2.0	Cellulose synthase A-catalytic [UDP-forming]
	Highest significantly expressed genes (more than four folds higher than control)					
	g3153		AphC/TSA family	g11772	4.2	Elongation Factor [TSF]
	g3480		AphC/TSA family	g5817	4.5	ACT-domain containing protein [ACR11]
	g3682		Intercellular signal transduction	g8657	4.2	Proline rich protein [DC2]
				g9421	5.5	Protein like isoform (plant invvertase/ pectin methylesterase inhibitor)
D6 vs S6	Salt stress defence mechanism related genes					
	g1120		GID1- like gibberellin receptor	g5647	1.0	Alpha/beta hydrolase (carboxylesterase)
	g802		GID1- like gibberellin receptor	g5851	1.2	Cinnamoyl-Co-A reductase-like
	Genes involved in physiological processes related to response to salt stress					
	g15315		Cysteine rich receptor-like protein kinase-2	g1047	1.8	Plastid glutamine synthase
	g243		ABA-insensitive like protein (bZIP1-TF)	g11093	1.4	Cellulose synthase A-catalytic [UDP-forming]
	g3644		ABA-insensitive like protein (bZIP1-TF)	g11199	1.5	Cellulose synthase A-catalytic [UDP-forming]
	g7343		EID-like F-box protein-3	g11449	1.5	Cellulose synthase A-catalytic [UDP-forming]
	g9718		Putative low temperature and salt responsive protein isoform-1	g146	1.5	Dicarboxylate transporter-1, chloroplast
				g16196	1.5	Dicarboxylate transporter-1, chloroplast
				g16418	1.7	Dicarboxylate transporter-1, chloroplast
				g16788	1.3	Dicarboxylate transporter-1, chloroplast
				g2795	1.0	Serine-glyoxylate aminotransferase
				g3130	1.1	Serine-glyoxylate aminotransferase
				g4724	1.1	Uncharacterized protein sequence
				g7120	1.1	Serine-glyoxylate aminotransferase
				g7168	1.9	Chlorophyll a-b binding protein CP2410-A
				g7762	1.2	Serine-glyoxylate aminotransferase
	Highest significantly expressed genes (more than four folds higher than control)					
	g10431		Energy production and conservation	g4007	5.4	Magnisium-chelatase [ChlH], Chloroplast
	g16087		Energy production and conservation	g4581	4.6	Alpha expansin
	g16288		Energy production and conservation			
	g2278		Energy production and conservation			
	g2618		Signal transduction mechanisms			
	g2863		Signal transduction mechanisms			
	g675		reverse transcription			
	g7343		EID-like F-box protein-3			
	g9017		Energy production and conservation			

After 6 h a number of 479 unigenes were significantly up-regulated, these genes belong to 45 different protein families and most of these families are involved in stress tolerance or defense mechanisms and metabolism, etc. Between these 479 unigenes there were 5 unigenes directly related to salt stress including bZIP-8 transcription factor, EID1-like F-box protein-3, WCOR-413 like cold acclimation protein and putative low temperature and salt responsive protein isoform with fold change values 1.8, 1.9, 3.6, 1.6 and 2.1 higher than control. Furthermore, among all significantly expressed genes there were 9 genes which gave the highest expression level. These nine genes were included under three different protein families i.e.*,* malate synthase, glyoxysomal; protein TRANSPARENT TESTA-12, detoxification; SNF-1 related protein kinase and two dehydrin unigenes (Table 4).

While, there were 567 unigenes significantly down-regulated at 6 h of salt stress. Among these genes there were 15 salt stress response unigenes belonging to different protein families including plastid glutamine synthase, Cellulose synthase A-catalytic (UDP-forming), decarboxylase transporter-1 (chloroplast), Indole-3 acetic acid amino synthase (GH3), tubulin alpha-2 chain-like, protein WALLSARE THIN-1 like and aquaporin protein-12. The highest down regulated unigenes (4 folds lower than control) were two unigenes belong to nitrate reductase (NADH) protein family (Table 4).

At 12 h of salt stress, a number of 281 unigenes were significantly expressed and up-regulated which belong to 32 different gene families. Between these genes there were 5 genes directly respond to salt stress treatment which were aligned to BEL-1 like homes domain protein-1 (BLH-1), bZIP-8 transcription factor and EID-1 like F-box protein-3. Moreover, the superior expressed genes were belonged to nucleoredoxin2 isoform X-1 (AhpC/TSA gene family). On the other hand, a number of 301 unigenes were significantly down-regulated including six unigenes involved in response to salt stress. These 6 unigenes belong to four different gene families i.e.*,* cellulose synthase A catalytic (UDP forming), Tubulin alpha-2 chain-like isoform × 2 and enol-[acyl-carrier-protein] reductase (NADH)- chloroplast-like. Furthermore, four unigenes were expressed four folds higher than control which included under four different gene families (elongation factor [TSF], ACT-domain containing protein [ACR-11], proline rich protein and protein like isoform and pectin methyl-esterase inhibitor (Table 4).

There were 508 unigenes significantly up-regulated in leaf tissues after 48 h of salt stress belonging to 63 different gene families. The unigenes which responded to salt stress were included under Cystein-rich receptor-like protein kinase-2, bZIP-8 transcription factor, EID-1 like F-box protein-3 and low temperature and salt responsive protein isoform. Furthermore, the highest expressed genes in leave tissues were belong to importin-5 malate synthase, glyoxysomal, protein phosphatase 2C-37 like, O-acyltransferase WSD-1-like, EID1-like F-box protein-3 and NADH-dehydrogenase. In addition there were 1335 significantly down-regulated unigenes at 48 h of salt stress. Furthermore, a number of 14 unigenes were directly involved in response to salt stress included under plastid glutamine synthase-2, cellulose synthase A- catalytic [UDP-forming], dicarboxylate transporter-1 [chloroplast], serine-glycosylate aminotransferase, chlorophyll a-b binding protein CP2410-A and one uncharacterized protein sequence. Out of the significantly down-regulated unigenes there were two unigenes which showed more pronounced effect include unigene4007 and 4581 which have been identified as Magnesium-chelatase [ChlH] in chloroplast and alpha expansin protein (Table 4).

As shown in Fig. 5 there were 119 up-regulated genes present in various durations of salt stress (6, 12, 48 h). Out of these 119 unigenes, 92 genes were significantly expressed at 6, 12, 48 h of salt stress. Pfam results showed that among these 92-salt induced genes, 77 unigenes (83.69%) were found to have a known function. A number of 5 unigenes (5.43%) out of the significantly expressed genes showed no homology with known sequence. Furthermore, three unigenes belong to bZIP-8 transcription factor and EID-1 like F-box protein-3 were significantly expressed in leave tissues and involved in response to salt stress at 6, 12, 48 h of salt stress. On the other hand, there were 87 down-regulated unigenes in leave tissues during 6, 12, 48 h of salt stress and out of these 87 unigenes there were 42 significantly down-regulated as compared to control (Fig. 6).

Based on the above mentioned results, all of these significantly unigenes during the three time points of salt stress (6, 12, 48 h) definitely have a role under salt stress conditions or may be contribute in regulating the salt response in Xuzi-8 sweetpotato cultivar.

### Differential expression of transcription factors (TFs) regulated by salt stress

There were 4, 1202, 764 and 2195 transcription factors differentially regulated DEGs by salt stress at different time points including 1, 6, 12, 48 h of salt stress. During the first hour of salt stress there were 4 different TFs up-regulated in the salt treated plants which were included under SBP, LIM, NAC and FAR1 TFs families.

At 6 h of salt stress the number of TFs increased giving 1202 TFs including 529 up-regulated and 672 down-regulated TFs. The most pronounced TFs regulated after 6 h of salt stress (expressed 5 folds than control) was aligned to malate synthase which is responsible for energy production and conversion. Followed by 9 unigenes expressed with 4 folds higher than control which were included under mTERF and WRKY TFs families. Furthermore, 38 unigenes belongs to NF-Y- > NF-YC, C2C2- > C2C2-GATA, DBP, C3H, WRKY, Tify, SNF2 and HB- > HB-HD-ZIP TFs families were up-regulated with 3 folds than control. On the other hand 672 TFs down regulated in leaf tissues of treated plants lower than control. The lowest down regulated unigenes were belonged to C3H and SET TFs families (namely: Nitrate reductase [NADH] and Auxin-binding protein) which are involved in oxidation-reduction process and auxin-activated signalling pathway, respectively. Unigenes belong to GNAT TF family including Aquaporin PIP2–7 and Chlorophyll a-b binding protein 48 were down regulated as compared to control. GNAT TF family are involved in Carbohydrate transport and metabolism, water transport and water channel activity. MADS- > MADS-M-type TF family aligned unigenes (down-regulated with 3 folds lower the control) are involved in lipid transport and metabolism.

After 12 h of salt stress, a number of 673 TFs were differentially regulated in treated plants than control plants including 341 up-regulated and 422 down-regulated TFs. The up-regulated TFs were aligned to different TF families including HB- > HB-HD-ZIP (13 unigenes), C2C2- > C2C2-GATA (12 unigenes), DBP (11 unigenes), SNF2 (10 unigenes), bZIP and C2H2 (8 unigenes), NF-Y- > NF-YC (6 unigenes) and WRKY (5unigenes). The highest up-regulated TFs (4 folds higher than control) was aligned to C2C2- > C2C2-GATA family namely nucleoredoxin 2 including Thioredoxin-like domain which is involved in intracellular signal transduction. Followed by DBP, bZIP and SNF2 TFs families including genes nucleoredoxin 2, protein phosphatase 2C, ABSCISIC ACID-INSENSITIVE 5-like protein 5 (bZIP8) and EID1-like F-box protein, respectively. On the other hand, there were 422 unigenes down regulated were classified as TFs including AP2/ERF- > AP2/ERF-ERF (31 unigenes), MYB- > MYB-related (22 unigenes), GNAT (15unigenes), C3H (13 unigenes), LIM (11 unigenes), Trihelix (11unigenes) and SWI/SNF-BAF60b (10 unigenes). MYB- > MYB-related (ACT domain-containing protein ACR11) and Trihelix (14 kDa proline-rich protein DC2.15-like) TFs families were the lowest expressed among all. In addition there were a number of 14 unigenes significantly down-regulated 3 folds less than control which were aligned to geraniol 10-hydroxylase-like protein, Elongation factor Ts, cytochrome P450, Expansin-A1, photosystem II protein D2, abscisic acid receptor PYL4-like, 14 kDa proline-rich protein DC2.15-like and gibberellin induced protein. These genes were belonged to HB- > HB-other, bHLH, LIM, MYB, Trihelix and mTERF TF families.

The number of TFs induced by exposure to 48 h of salt stress was the highest among all time points (2194 TFs) including 663 up-regulated and 1531 down-regulated unigenes. The up-regulated TF families were C3H (26 unigenes), WRKY (16 unigenes), bZIP (15unigenes), NAC (15 unigenes), FAR1 (15 unigenes), HB- > HB-HD-ZIP (13 unigenes), PHD (13 unigenes), C2H2 (12 unigenes), SNF2 (11 unigenes), AP2/ERF- > AP2/ERF-ERF (11 unigenes) and B3- > B3 (11 unigenes). On the contrary, there were 1531 down regulated TFs included under different TFs families. One unigene encoded magnesium-chelatase subunit ChlH (chloroplastic) was expressed five folds lower than control was belonging to Jumonji TF family which is involved in chlorophyll biosynthetic process and work as a coenzyme transport and metabolism. A unigene aligned to Expansin-A2 belongs to LIM TFs family was down-regulated 4 folds lower than control which found to be plant-type cell wall organization. In addition, there were 33 unigene expressed with 3 folds lower than control.

### Protein kinases (PKs) differentially expressed under salt stress

In sweetpotato at the time of salt stress, PKs include many genes and play a vital role in phosphorylation process and act as a signal transductor/receptor proteins in membranes.

SNF1-related protein kinase was up-regulated during 6 h (7 unigenes) and 48 h (4 unigenes) of salt stress expressed in two forms including SNF1-related protein kinase catalytic subunit alpha KIN10-like and SNF1-related protein kinase regulatory subunit gamma-1. One unigene aligned to Phosphatidylinositol 4-phosphate 5-kinase was up-regulated during 6 and 48 h after salt stress. A number of 19 unigenes were aligned to cyclin-dependent kinase which were up-regulated at 6 and 48 h of salt stress. CBL-interacting protein kinase 10 aligned genes were down-regulated. Cysteine-rich repeat secretory protein 3 is a salt stress response gene was up-regulated starting from 6 h of salt stress. G-type lectin S-receptor-like serine/threonine-protein kinase aligned genes (14 unigene) were up-regulated only at 12 h of salt stress. A number of 6 unigenes aligned to wall-associated receptor kinase were down-regulated starting from 6 till 48 h of salt stress. A number of 46 unigenes were classified under LRR receptor-like protein kinase were up-regulated at 12 and 48 h of salt stress.

### Photosynthesis related genes under salt stress

Leaf is the factory of the plant that provide the plant with necessary energy for different physiological processes through photosynthesis. Genes related to photosynthetic parameters including gas exchange, pigments are affected seriously by salt stress. The current transcriptome sequencing results showed that there were 441 unigenes identified to be involved in photosynthetic related processes or pathways. Exposing he plant for 200 mM of Nacl for 1 h didn’t significantly affect photosynthesis process or its related genes. Most of genes involved in photosynthesis process were down regulated including 88, 20 and 239 DEGs at 6, 12, 48 h of salt stress as compared to control. While, a very low number of DEGs were up-regulated including 8 and 5 DEGs at 6 and 48 h of salt stress as compared to control. At 6 h of salt stress a number of 8 unigene aligned to Fructose-1,6-bisphosphatase (cytosolic) were up-regulated. Furthermore, after 48 h there were only 5 up-regulated DEGs which were aligned to Aconitate hydratase (cytoplasmic) and heme oxygenase 1 (chloroplastic). In addition there was one gene was up-regulated during 6 and 48 h of salt stress which is encoded to stress enhanced protein 2 (chloroplastic). On the other hand, there were 10 unigenes down regulated at 6, 12, 48 h which were aligned to ADP-glucose pyrophosphorylase beta, thylakoid lumenal 29 kDa protein (chloroplastic), chlorophyll a-b binding protein (chloroplastic) and rhodanese-like domain-containing protein 9 (chloroplastic). At 6 h the most pronounced down-regulated gene as compared to control was chlorophyll a-b binding protein which is common in all time points beside Protochlorophyllide reductase (chloroplastic). At 12 h beside to the common genes, serine hydroxymethyltransferase (mitochondrial), mitogen-activated protein kinase, photosystem II protein D2, Photosystem I reaction center subunit XI (chloroplastic) and NAD(P)H-quinone oxidoreductase (chloroplastic) were down regulated. At 48 h the lowest down-regulated gene was magnesium-chelatase subunit ChlH (chloroplastic) followed by chlorophyll a-b binding protein and photosystem I reaction center subunit XI (chloroplastic).

### SSR and SNP identification

**Fig. 7:**
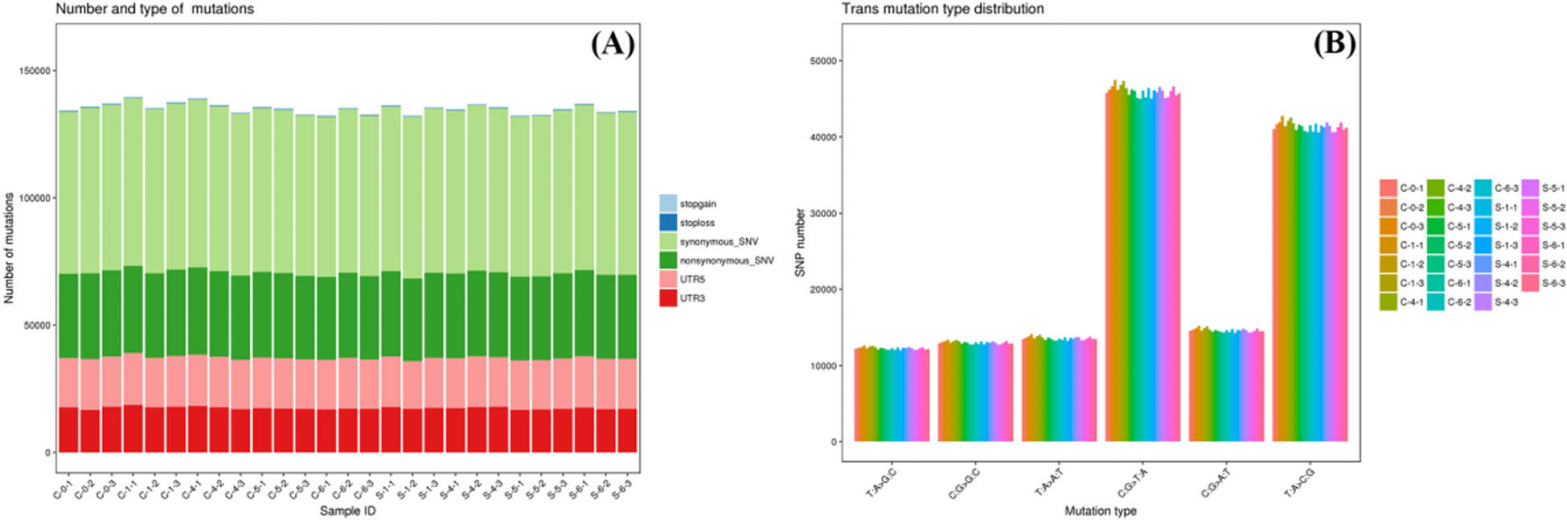
Simple sequence repeat (SSRs) and single nucleotide polymorphism (SNPs) detected in the obtained sequences in Xuzi-8 sweetpotato cultivar under salt stress conditions (200mM NaCL) with 5 libraries (0, 1, 6, 12, 48 hours). **a**; Frequency and distribution of SSRs in coding sequence and untranslated region (UTRs). **b**; Frequency and substitution types of the identified SNPs in the five libraries (three replicates per each library)

For further application of sweetpotato SSRs and SNPs were discovered using assembled transcriptomes (Fig. 7a and b). A total of 24,559 SSRs were identified in transcriptomes present in 15,976 sequences. Furthermore, the numbers of sequence containing more than one SSR were 5762 and the numbers of SSRs present in compound formation were 2215. In addition, the major types of the identified SSRs were mono-nucleotide (19,380), di-nucleotide (2791), tri-nucleotide (2199), tetra-nucleotide (123), penta-nucleotide (33) and hexa-nucleotide (25). The most SSR motif was A/T (19282) followed by AG/CT (1964), AT/AT (669), AAG/CTT (596), CCG/CGG (330), AAT/ATT (309), ATC/ATG (247) and AGC/CTG (217). A total of 562,174 SNPs between transcriptomes were identified, among which 348,495 were transitions, and 213,679 were transversions. These SSRs and SNPs identified in this study provided a valuable resource for future studies on genetic linkage mapping and the analysis of interesting traits in sweetpotato.

### Experimental validation by qRT-PCR

**Fig. 8:**
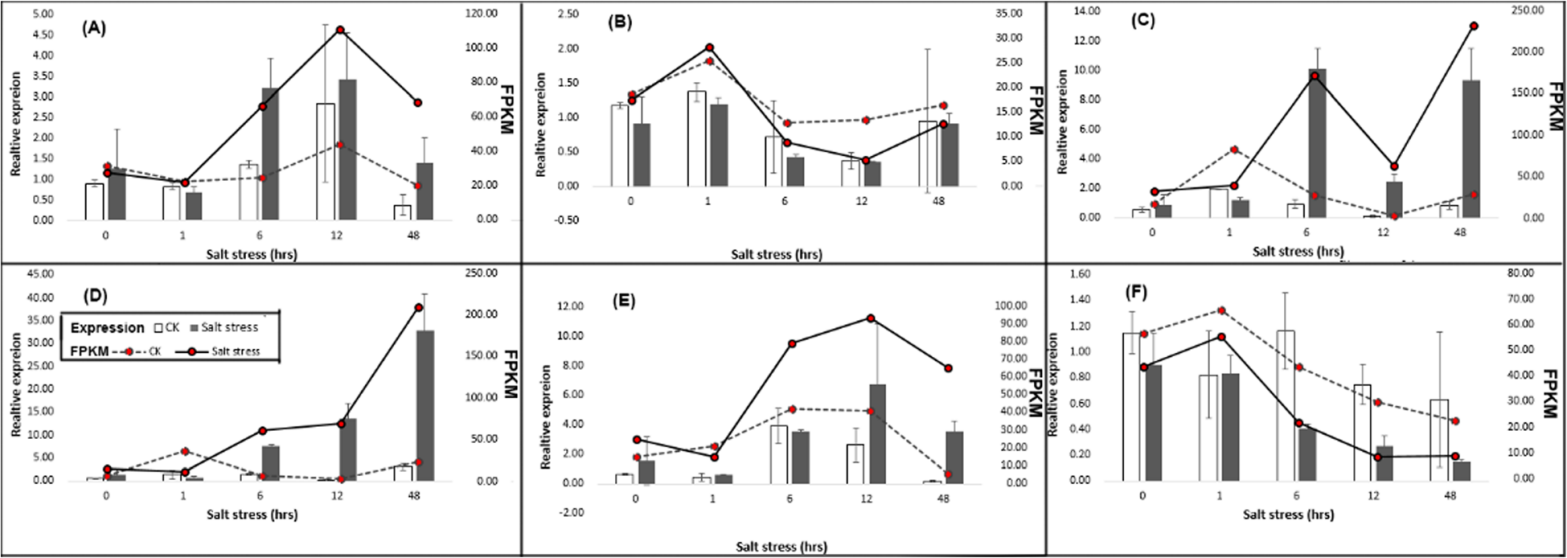
qRT-PCR validation of the salt induced fold changes detected in Xuzi-8 sweetpotato cultivar using RNA-seq. Standard error bars are showed for the expression values determined by qRT-PCR. The expression levels determined by qRT-PCR and DEG values of the genes (protein disulfide isomerase, Actin-12 (ACT12), nucleoredoxin 2 isoform X1, Thioredoxin-like domain, CTD small phosphatase-like protein 2 and Redoxinwere presented) at (**a**), (**b**), (**c**), (**d**), (**e**) and (**f**), respectively. Expression values determined by qRT-PCR are presented as columns and DEGs are presented as curves

To confirm the reliability of the illumine sequencing, six unigenes were randomly selected for quantitative RT-PCR assays including unigene299, 2083, 2359, 3153, 6075 and 6453. The results showed that the DEG results and expression levels have the same tendency among all unigenes (Fig. 8). The unigenes 299, 2359, 3153 and 6075 were up-regulated in both qPCR results and DEG analysis giving the lowest values at the first time point (1 h of salt stress) then started to increase till 48 h of salt stress. On the other hand, the unigenes 2083 and 6453 were down regulated with an expression level one fold lower than control samples which was in agree with the DEG results (Fig. 8a-f).

## Discussion

Sweetpotato is a hexaploid heterozygous non-model crop with a complex genome lacking high quality reference genome [13]. To date the current understanding of the complex physiological and molecular mechanisms of salt tolerance in sweetpotato remains limited [14, 15]. High throughput RNA sequencing is required for identification of candidate genes involved in salt stress tolerance. In addition, it will be helpful for better understanding of salts stress tolerance mechanisms in sweetpotato [5].

In the present study, deep sequencing analyses of PFSP under different durations of salt stress were characterized as compared to control. Using second and third generation technology, Illumina sequencing generated 170,344,392 clean high-quality long reads that were assembled into 15,998 unigenes with an average length 2178 base pair which considered relatively longer than only NGS studies which ranged between 1000 and 1300 base pairs as an average length for the obtained sequence [16–19]. A percent of 96.55% of the obtained unigenes were functionally annotated in the NR protein database, while there were 537 unigenes failed to hit any homologs which may be considered as new protein sequence which had not been characterized previously in sweetpotato.

The current results revealed that there are similarity with the available sweetpotato genome sequence at a percent of 3.07%. That’s mean that 96.93% of our obtained sequence are not present on the sweetpotato available online data. Therefore, the current results will contribute to improve sweetpotato genome annotation and facilitate the discovery of genetic resources that are responsible for salt stress in hexaploid sweetpotato.

The results in Fig. 3 showed that the most dominant Go-terms which were identified during the different durations of salt stress included metabolic, cellular, single organism and response to stimulants. These results are in agree with [20] in diploid halophytic sweetpotato, [21] in *Ipomoea imperati* [22]. Furthermore, COG results indicated that the highest number of genes was categorized as “post-translational modification, protein turnover, chaperones” in our study. Which mean that, sweetpotato plants under salt stress conditions, starts the key mechanisms of chemical modifications including regulating of enzymes activity, localization and interaction with other cellular molecules such as proteins, nucleic acid, lipids and co-factors [23]. Furthermore, protein turnover related genes control the balance between protein biosynthesis and degradation, which is very important for determining the resistance or sensitivity of the plant to salt stress. That’s may be due to if the protein biosynthesis is more than breakdown indicates an anabolic state, in this case the plant tolerance to stress will be enriched [24–27]. Moreover, Protein chaperones’ major function is to prevent or correct damage caused by miss-folding due to salt stress [28–31]. The second COG group of genes involved in “signal transduction mechanisms” which is mainly transforming the certain stimulus induced by salt stress into a biochemical signal which activates more genes specifically involved in salt stress tolerance [32].

### The regulatory mechanisms and salt resistance related genes in sweetpotato

From the molecular aspects, salt tolerance in sweetpotato starts when the plant exposed to salt stress, the action taken including activating the transcriptional control inside the nucleus of the cells, sensing and signaling, cellular influx and detoxification mechanisms activated [33].

TFs play an essential role in regulating many different signal transduction pathways in plants under stress by activating the expression of specific genes [34]. In the current study, the response of leaf tissues was lower during the first hours of salt stress and there were only four TFs significantly up-regulated with one fold higher than control. These four unigenes including SBP TFs have important role in leaf development, vegetative phase change and may be have a role in stress response [35]. The second unigene was aligned to HSP70 which is essential regulator to maintain internal cell stability and prevent aggregation under physical or chemical pressure [36]. The plant invertase/ pectin methyl-esterase inhibitor affects growth and development consisting with its activities such as stress response. In addition, pectin methyl-esterase determines the solidity of cell wall including root development and permeability [37]. These four genes considered as early response genes for stress in leaf tissues [38] which can improve stress tolerance at the first phase of stress. This indicate that Xuzi-8 sweetpotato as a tolerant cultivar slightly sensed osmotic stress at the first hour of salt stress. However, only four TFs were up-regulated which considered necessary in linking salt sensory pathways to salt tolerance as well as other types of stress.

In addition the current results showed that a core sets of TF family genes were differentially expressed in leaf tissues during 6, 12 and 48 h of salt stress including C3H, WRKY, bZIP, NAC, FAR1, HD-ZIP, PHD, C2H2, SNF2, AP2/ERF, MYB and B3. In agree with our results Geng Y. et al., [39] reported that most of stress induced transcriptional changes occurs at least after 3 h of salt stress exposure. These TFs in turn are regulating the expression levels of different genes that may ultimately enrich salt tolerance in sweetpotato [39].

In the current study, at 6 h of salt stress the most pronounced TFs were malate synthase, mTERF and WRKY. These three TFs have an important role in plant physiology under salt stress conditions [40]. Followed by SNF-1 related protein kinase (represent an interface between metabolic and stress signaling) [41] and dehydrin (play a major role in recovery of drought and salt stress) [42]. In addition to TFs, at 6 h, there were protein TRANSPARENT TESTA-12 which control flavonoid sequestration [43] and Protein DETOXIFICATION is delay the development of disorders associated with stress [43]. Furthermore, ABA insensitive-like protein is acting as a positive component in glucose signal transduction [43] and EID-like F-box protein family is a known drought and salt response regulation after 6 h. WCOR 413-like protein is responsible for protection of plasma membrane against dehydration which has been discovered earlier in sweetpotato genome, these findings are in agree with [43] in wheat. The putative salt stress responsive protein isoform-1, which play a critical role in salt stress tolerance after 6 h, has been discovered before in sweetpotato genome [44]. Thus, we can conclude that at 6 h the plant start to activate the TFs which regulate genes involved in salt stress tolerance, protein kinases that save the metabolic activity and activate stress signaling beside protein detoxifications which delay the harmful effect of salt stress and reduce the excess absorption of NaCL.

The highest regulated TFs at 12 h of salt stress as compared to control was nucleoredoxin 2 including Thioredoxin-like domain (AhpC/TSA TFs) which is responsible for signal transduction which in role constitutes an enzymatic defense against salt stress. In addition, at 12 h of salt stress, BEL-1 like homes domain protein-1 (BLH-1) contribute in regulating a range of developmental processes under salt stress, bZIP-8 transcription factor is a key components in response to a wide range of abiotic stresses, including high salinity through regulating ABA concentration. In addition ABA is responsible for preventing the lateral root elongation into surrounding media with high salt concentration. Furthermore, EID-1 like F-box protein-3 were expressed with high level as compared to control [43]. Therefore, at 12 h the genes involved in sensing and signaling were more expressed, beside the TFs which are involved in hormonal regulation which have a major role in defense mechanisms against salt stress.

In the current study, as compared to control, among the other time points, the number and expression level of TFs at 48 h was the highest. These TFs families which have a strong relation with salt stress tolerance at 48 h including C3H, WRKY, bZIP, NAC, FAR1, HB- > HB-HD-ZIP, PHD, C2H, SNF2, AP2/ERF- > AP2/ERF-ERF, EID-1 like F-box protein-3 and B3- > B3. In addition, the unigenes which responded to salt stress were Cystein-rich receptor-like protein kinase-2, low temperature and salt responsive protein isoform. Furthermore, the highest expressed genes in leave tissues were belong to importin-5 malate synthase, glyoxysomal, protein phosphatase 2C-37 like, O-acyltransferase WSD-1-like, and NADH-dehydrogenase [45–48].

### Photosynthesis related genes

Photosynthesis is the most significant physiological process for the plant life and during all growth stages is affected by stress factors [33]. In the current results, it was interesting to note that most of DEGs related to photosynthesis were down-regulated, while, a very low number of unigenes were up-regulated. The stress enhanced protein 2 (chloroplastic) was significantly up-regulated at 6 and 48 h of salt stress. In addition, fructose-1,6-bisphosphatase (cytosolic) was up-regulated after 6 h of salt stress which is involved in carbohydrate transport and metabolism and Aconitate hydratase (cytoplasmic) and Heme oxygenase 1 (chloroplastic) were up-regulated at 48 h of salt stress. According to our results, Heme oxygenase 1 (chloroplastic) was up-regulated which is responsible for inorganic ion transport and metabolism that’s in role counteract the reduction of water potential resulting from osmotic components of enhanced salinity [39]. On the other hand, the number of genes related to photosynthesis was 88 at 6 h off salt stress decreasing into only 20 unigenes at 12 h and reached to the maximum at 48 h of salt stress. There were 10 unigenes common in the three time points 6, 12, 48 h including ADP-glucose pyrophosphorylase beta, thylakoid lumenal 29 kDa protein (chloroplastic), chlorophyll a-b binding protein (chloroplastic) and rhodanese-like domain-containing protein 9 (chloroplastic). Furthermore, the down-regulated unigenes were involved in amino acid transport and metabolism, carbohydrate transport and metabolism, cell wall/membrane/envelope biogenesis, secondary metabolites biosynthesis, transport and catabolism, energy production and conversion, inorganic ion transport and metabolism and coenzyme transport and metabolism. To some extent, genes involved in the above mention physiological processes are mediated by dynamic changes in photosynthesis [39]. Therefore, according to hour results and in agree with previous researches, photosynthesis process severely affected by salt stress starting from 6 h and the effect increase with increasing the stress duration.

Taken together, TFs (bHLH, bZIP, C2H2, C3H, C3H4, ERF, MYB, NAC, TSA and WRKY) regulates the differentially expressed genes that related to salt stress tolerance and PKs act as a signal transductor/receptor proteins in membranes. Furthermore, protein detoxifications have damage control and repair associated with stress. In addition genes related to hormonal balance have a major role in determining the level of plant tolerance. According to our results, due to the high expression of these genes including TFs, PKs, Protein Detox and hormones related genes enriched the salt tolerance in Xuzi-8 sweetpotato cultivar.

## Conclusions

In the present study, de novo was constructed and characterized the transcriptomes of sweetpotato challenged with salt stress and identified 15,976 unigenes, generating a broad survey of genes involved in salt stress resistance. DEG profiling analysis at the significance level identified number of 4, 479, 281, 508 up-regulated and 0, 567, 301, 1335 down-regulated unigenes. Functional analysis of the obtained sequence showed that the main TFs families involved salt stress tolerance including bHLH, bZIP, C2H2, C3H, C3H4, ERF, MYB, NAC, TSA and WRKY. PKs act as a signal transductor/receptor proteins in membranes and protein detoxifications have damage control and repair associated with stress. The current transcriptome sequencing data of hexaploid sweetpotato under salt stress conditions can provide a valuable resource for sweetpotato breeding research and focus on novel insights into sweetpotato responses to salt stress. In addition, it offers new candidate genes or markers that can be used as a guide to the future studies attempting to breed salt tolerance sweetpotato cultivars.

## Methods

### Plant materials

Xuzi-8, a high quality, early mature cultivar with green leaves and purple flesh storage roots, and salt tolerance, was used in this experiment. Its storage roots contain 6% soluble sugar and more than 80 mg anthocyanin /100 g fresh weight. It have a strong vegetative growth and its leaves contain 398.31, 130.75 and 39.27 mg/100 g dry weight of chlorophyll a, chlorophyll b and carotenoids, respectively [49]. This cultivar was obtained from Xuzhou Institute of Agricultural Sciences in Jiangsu Xuhuai District, China.

### Treatments and experimental design

Apical stem cuttings (15–20 cm) of Xuzi-8 cultivar taken from 2 months age healthy sweetpotato plants growing in the field. Stem cuttings were let to grow in hydroponics culture using Hoagland nutrient medium till having 1 cm pencil roots (about 3 weeks). Seedlings were exposed to salt stress (200 mM NaCl were added to Hoagland solution) for 0, 1, 6, 12, 48 h which were chosen according to our previous study [50]. 0.1 g sample (three replicates per each sample) was taken from the upper third leaf after 0, 1, 6, 12, 48 h from salt treated and untreated plants for the next generation sequencing. For the third-generation sequencing, samples was collected from the upper third leaf at the same time points (three replicates for each sample). All collected samples were immediately frozen in liquid nitrogen and stored at − 80 °C until processing for RNA extraction and all treatments were done in triplicate.

### RNA extraction

Total RNA was extracted from collected samples with the TRIZOL method (Life technologies, Carlsbad, CA) according to the manufacturer’s protocol. RNA degradation and contamination were monitored on 1% agarose gels. RNA purity was checked using the Nanophotometer® spectrophotometer (IMPLEN, CA, USA). RNA concentration was measured using Qubit® RNA Assay Kit in Qubit® 2.0 Fluorometer (Life Technologies, CA, USA). RNA integrity was assessed using the RNA Nano 6000 Assay Kit of the Agilent Bioanalyzer 2100 system (Agilent Technologies, CA, USA).

### Library preparation for Transcriptome analysis

For second generation sequencing, a total amount of 3 μg RNA per sample was used as input material for the RNA sample preparations. Sequencing libraries were generated using NEBNext® Ultra™ RNA Library Prep Kit for Illumina® (NEB, USA) following manufacturer’s protocol and index codes were added to attribute sequences to each sample. For third generation sequencing RNA was extracted from different time points and equal amount of each sample were mixed to produce one library beside to the control library. Briefly, mRNA was purified from total RNA using poly-T oligo-attached magnetic beads. Fragmentation was carried out using divalent cations under elevated temperature in NEBNext First Strand Synthesis Reaction Buffer(5X). First strand cDNA was synthesized using random hexamer primer and M-MuLV Reverse Transcriptase (RNase H-). Second strand cDNA synthesis was subsequently performed using DNA Polymerase I and RNase H. Remaining overhangs were converted into blunt ends via exonuclease/polymerase activities. After adenylation of 3′ ends of DNA fragments, NEBNext Adaptor with hairpin loop structure were ligated to prepare for hybridization. In order to select cDNA fragments of preferentially 150~200 bp in length, the library fragments were purified with AMPure XP system (Beckman Coulter, Beverly, USA). Then 3 μl USER Enzyme (NEB, USA) was used with size-selected, adaptor-ligated cDNA at 37 °C for 15 min followed by 5 min at 95 °C before PCR. Then PCR was performed with Phusion High-Fidelity DNA polymerase, Universal PCR primers and Index (X) Primer. At last, PCR products were purified (AMPure XP system) and library quality was assessed on the Agilent Bioanalyzer 2100 system.

### Clustering and sequencing

The clustering of the index-coded samples was performed on a cBot Cluster Generation System using TruSeq PE Cluster Kit v3-cBot-HS (Illumia) according to the manufacturer’s instructions. After cluster generation, the library preparations were sequenced on an Illumina platform and paired-end reads were generated.

### Data analysis quality control

Raw data (raw reads) of fastq format were firstly processed through in-house perl scripts. In this step, clean data (clean reads) were obtained by removing reads containing adapter, reads containing ploy-N and low-quality reads from raw data. At the same time, Q20, Q30, GC-content and sequence duplication level of the clean data were calculated. All the downstream analyses were based on clean data with high quality.

### Transcriptome assembly

Transcriptome sequencing was accomplished based on both NGS and 3rd GS, and TPM, FPKM, RPKM and fold change (FC) were recorded for each replicate of each library separately. Obtained sequence from NGS and 3rd GS was aligned and similar sequence data from all libraries/samples were pooled [51] and ultimately used for further analysis using Trinity [52] with min_kmer_cov set to 2 by default and all other parameters set default.

### Gene functional annotation

Gene function was annotated based on the following databases: Nr (NCBI non-redundant protein sequences, https://www.ncbi.nlm.nih.gov/) with E-value cut-off of le-5; Nt (NCBI non-redundant nucleotide sequences https://www.ncbi.nlm.nih.gov/) with E-value cut-off of le-5 ; Pfam (Protein family, http://pfam.sanger.ac.uk/) with E-value cut-off of le-2; KOG/COG (Clusters of Orthologous Groups of proteins, https://www.ncbi.nlm.nih.gov/cog/) with E-value cut-off of le-3 and Swiss-Prot (A manually annotated and reviewed protein sequence database, http://www.ebi.ac.uk/uniprot/) with E-value cut-off of le-5.

Based on the NR and Pfam annotations, Blast2GO (v2.5) was used to obtain GO (Gene Ontology) annotations (http://www.geneontlology.org) according to the molecular functions, biological processes and cellular component ontologies [53]. GO enrichment analysis of the differentially expressed genes (DEGs) was implemented by the GO-seq R packages based Wallenius non-central hyper-geometric distribution [54], which can adjust for gene length bias in DEGs. KOBAS software (http://www.genome.jp/kegg/) were used to test the statistical enrichment of DEGs in KEGG [55].9. SNP calling and SSR detection.

Picard - tools v1.41 and samtools v0.1.18 were used to sort, remove duplicated reads and merge the bam alignment results of each sample. GATK software was used to perform SNP calling. Variants were kept for quality using the following parameters (1) mapping quality filter equal to PASS; (2) Quality Depth (QD) > 2; (3) Mapping Quality (MQ) > 40; (5) QUAL > 30; Moreover, variants were further filtered if coverage < 10, if cluster SNPs more than 2 in 5 bp window, if SNP around Indel within 5 bp.

SSR of the transcriptome were identified using MISA (http://pgrc.ipk-gatersleben.de/misa/misa.html), and primer for each SSR was designed using Primer3 (http://primer3.sourceforge.net/releases.php).

### Quantification of gene expression levels and differential expression analysis

Gene expression levels were estimated by RSEM [56] for each sample. Clean data were mapped back onto the assembled transcriptome. Read count for each gene was obtained from the mapping results.

Differential expression analysis of two conditions/groups was performed using the DESeq2R package DESeq2 rovide statistical routines for determining differential expression in digital gene expression data using a model based on the negative binomial distribution. The resulting *P* values were adjusted using the Benjamini and Hochberg’s approach for controlling the false discovery rate. Genes with an adjusted *P*-value ≤0.001 found by DESeq2 were assigned as differentially expressed [57]. Q-value≤0.001& |log2FC(foldchange)| ≥ 1 was set as the threshold for significantly differential expression.

### Quantitative and real time (qRT-PCR) validation

To confirm the reliability of the illumine sequencing, six unigenes were randomly selected for quantitative RT-PCR assays. Total RNA was extracted from collected samples with the TRIZOL method (Life technologies, Carlsbad, CA) and reverse transcription were done according to the manufacturer’s protocol. RT-qPCR was performed using the method described by [58] and Ubi3 (AY486137.1) was used as internal control (reference gene) [59]. qPCR primers of each unigene were designed using primer 5 software are shown in Table 5. All reactions were prepared in triplicates and relative expression was calculated using 2^(−∆∆Ct) method with the expression normalised against the internal reference gene (Tubulin2) [60].

**Table 5:** RT-qPCR primers for validation experiment

Unigene code	Direction	Sequence (5'->3')	Length
g299	Forward	TTTTCGTAATCCTGGCGGCG	20
	Reverse	ACCGTGTCGGAGAAGTTGGT	20
g2083	Forward	CCGCCCGAGAGGAAGTACAG	20
	Reverse	GGGCCAGACTCGTCGTACTC	20
g2359	Forward	GGAGGCGTTTGCTGCTTACC	20
	Reverse	TGCCCTCGACGTTGAACCTT	20
g3153	Forward	TTCGACGCCTACTTCGGGAC	20
	Reverse	GTCCGGCCCGAGAATTACCA	20
g6075	Forward	TCTAGCAATGGTGCTGCGGA	20
	Reverse	AGCCCTACTGCTCCAACTACG	21
g6453	Forward	TATGGTGTCGGTACGGCGTC	20
	Reverse	TGACTCAGTTCTAGCGGCCC	20

## Supplementary information

**Additional file 1.** GO function annotations including number of unigenes and unigenes ID; COG classes and functions with unigenes ID.

**Additional file 2.** Detailed GO function classification of differentially expressed unigenes between control and different treatments.

**Additional file 3.** The Most enriched GO terms during the different time points as compared to the control.

**Additional file 4.** KEGG different pathways including unigenes belonging for different pathways.

## Data Availability

Not applicable.

## Abbreviations

3rd GS: Third generation sequencing
FDR: False discovery rate
FPKM: Fragments per kilo base million
GO: Gene ontology
KOG/COG: Clusters of orthologous groups of proteins
NGS: Next generation sequencing
Nr: NCBI non-redundant protein sequences
Nt: NCBI non-redundant nucleotide sequences
PFSP: Purple flesh sweetpotato
RPKM: Reads per kilo base million
TPM: Transcripts per kilo base million
