# Supplementary material for: Transcriptome sequencing and whole genome expression profiling of hexaploid sweetpotato under salt stress
Source: BMC Genomics. 2020 Mar 4;21:197. doi: 10.1186/s12864-020-6524-1 (PMC7057664; doi:10.1186/s12864-020-6524-1)
Supplement: Supplementary file 2 — Additional file 2. Detailed GO function classification of differentially expressed unigenes between control and different treatments. [file 12864_2020_6524_MOESM2_ESM.docx]

Additional file 2: Figure S1. An overview of GO function classification of differentially expressed unigenes between control and different treatments


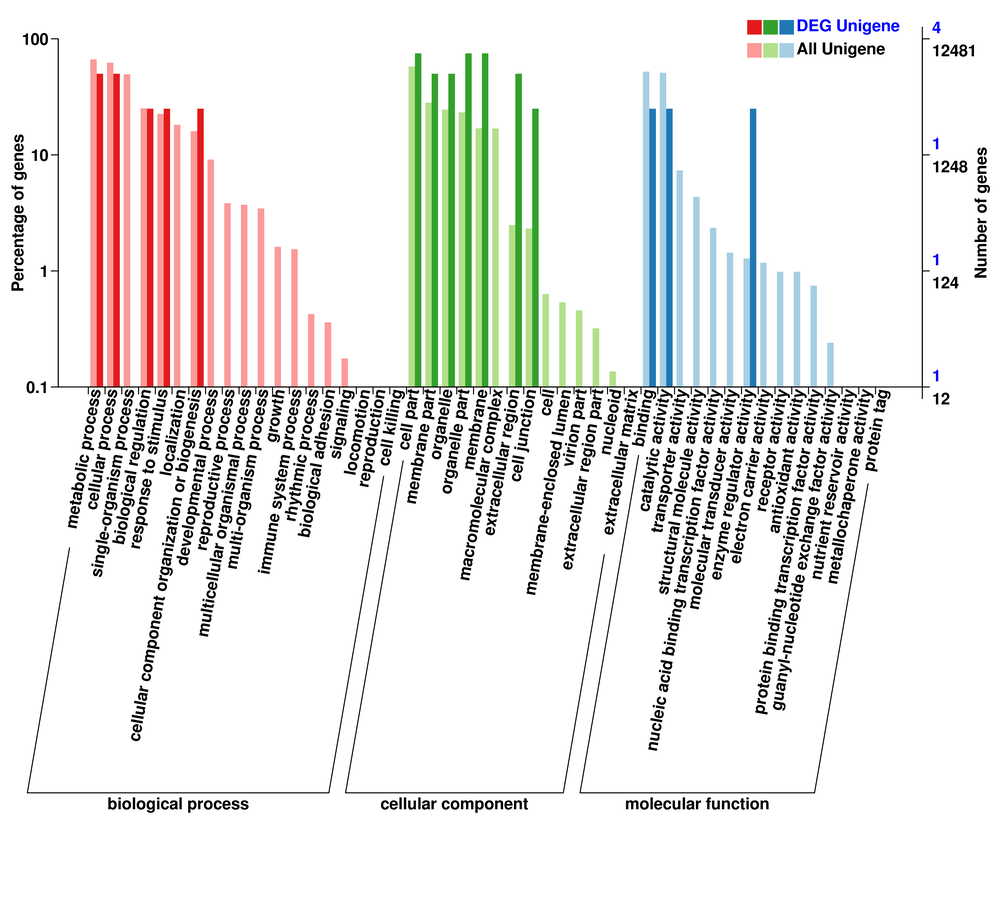


0hr vs 6 hrs - GO classification

0hr vs 1 hr GO classification


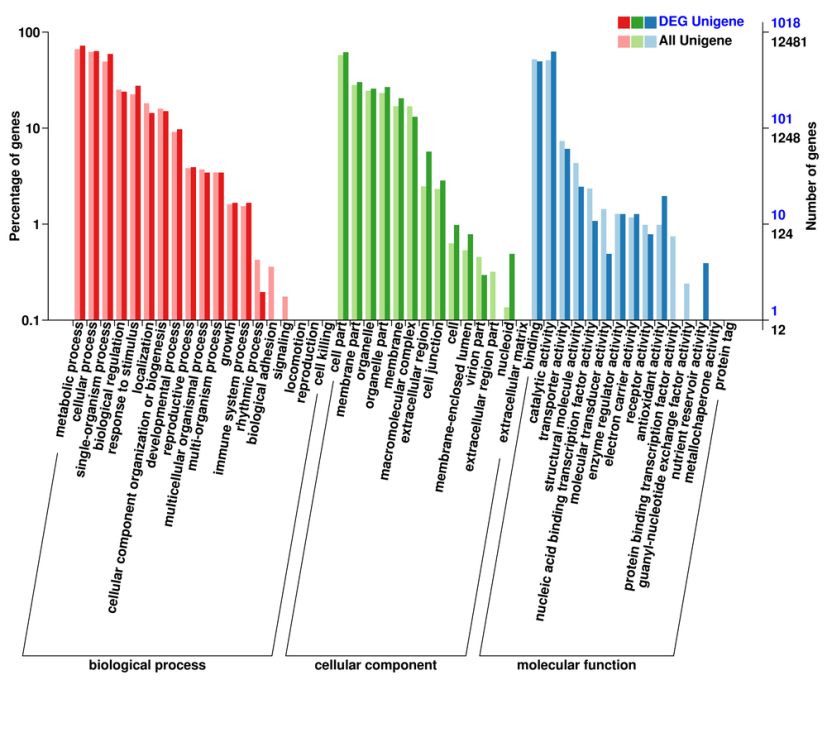


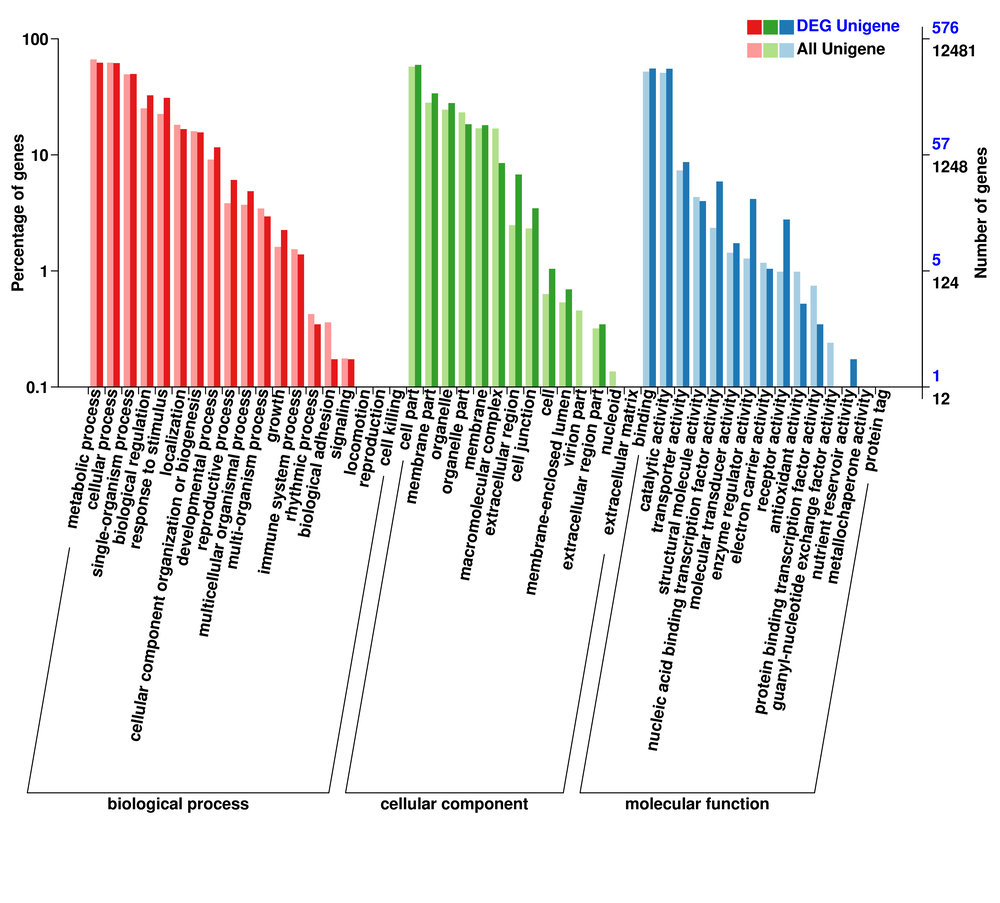


0hr vs 12 hrs - GO classification


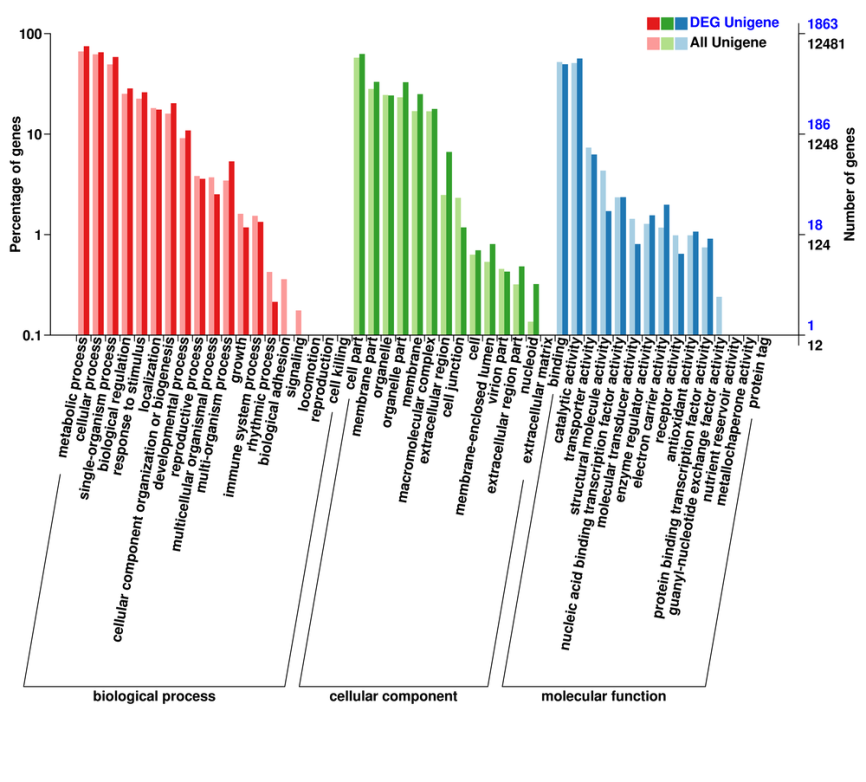


0hr vs 48 hrs - GO classification
