# Supplementary material for: Transcriptome sequencing and whole genome expression profiling of hexaploid sweetpotato under salt stress
Source: BMC Genomics. 2020 Mar 4;21:197. doi: 10.1186/s12864-020-6524-1 (PMC7057664; doi:10.1186/s12864-020-6524-1)

Additional File 3: The Most enriched GO terms during the different time points as compared to the control


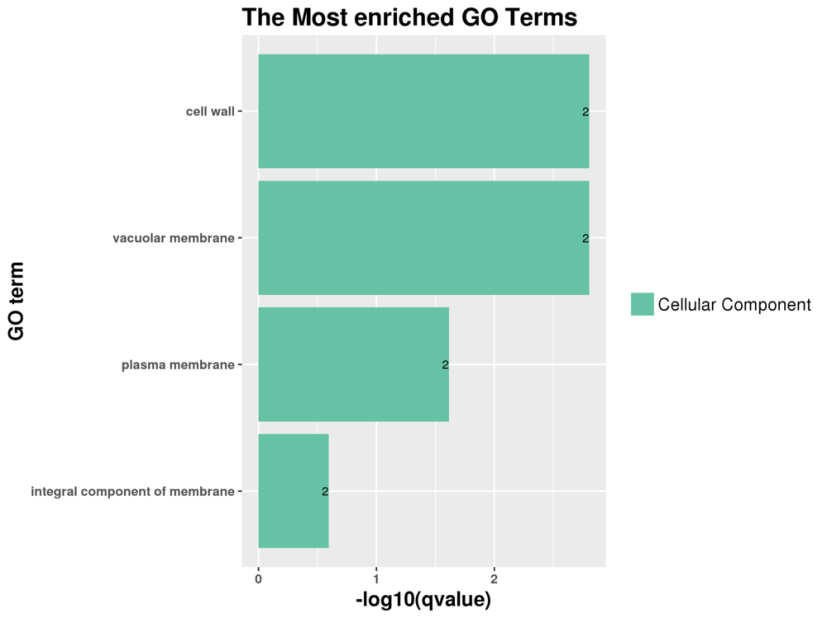


0hr vs 1hr

0hr vs 6hr


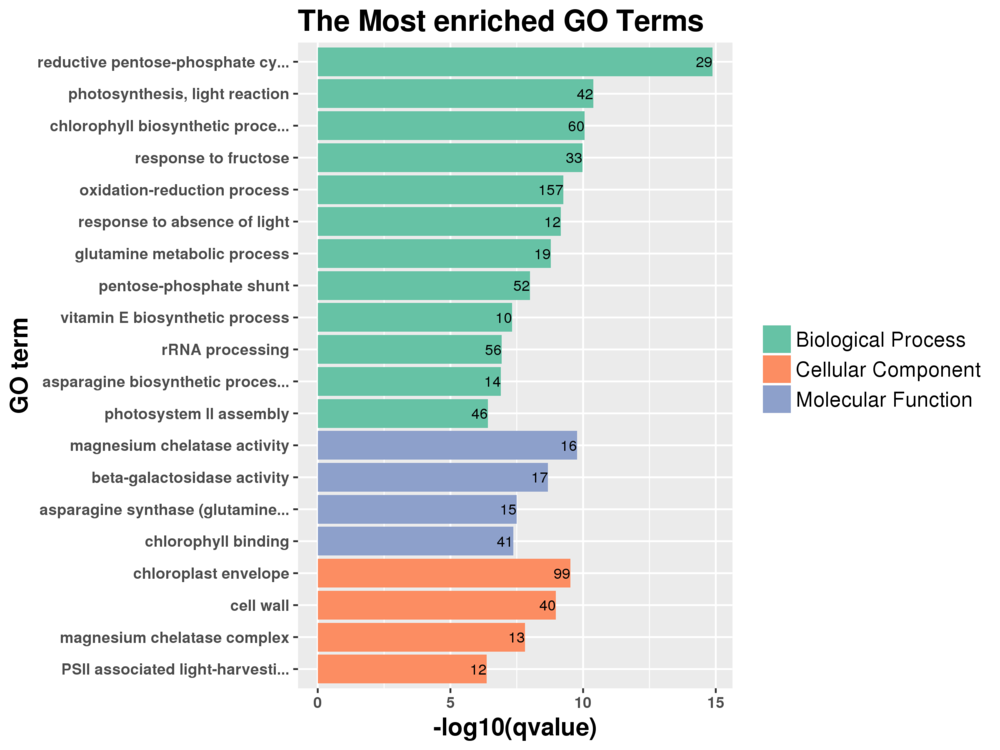


0hr vs 12hrs


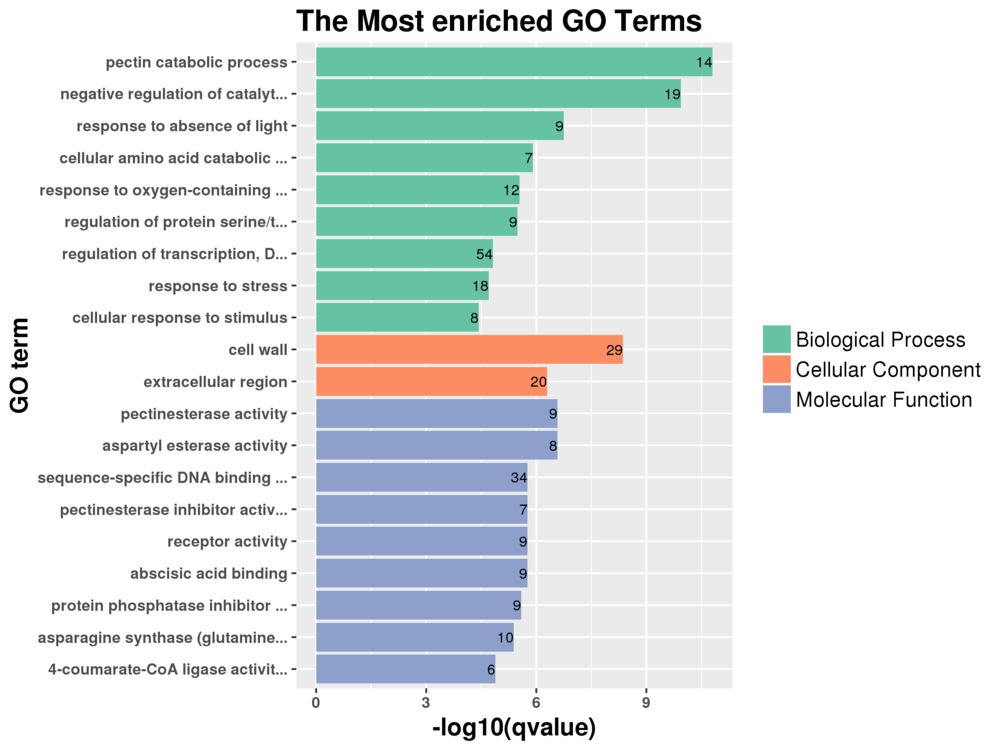


0hr vs 48hrs


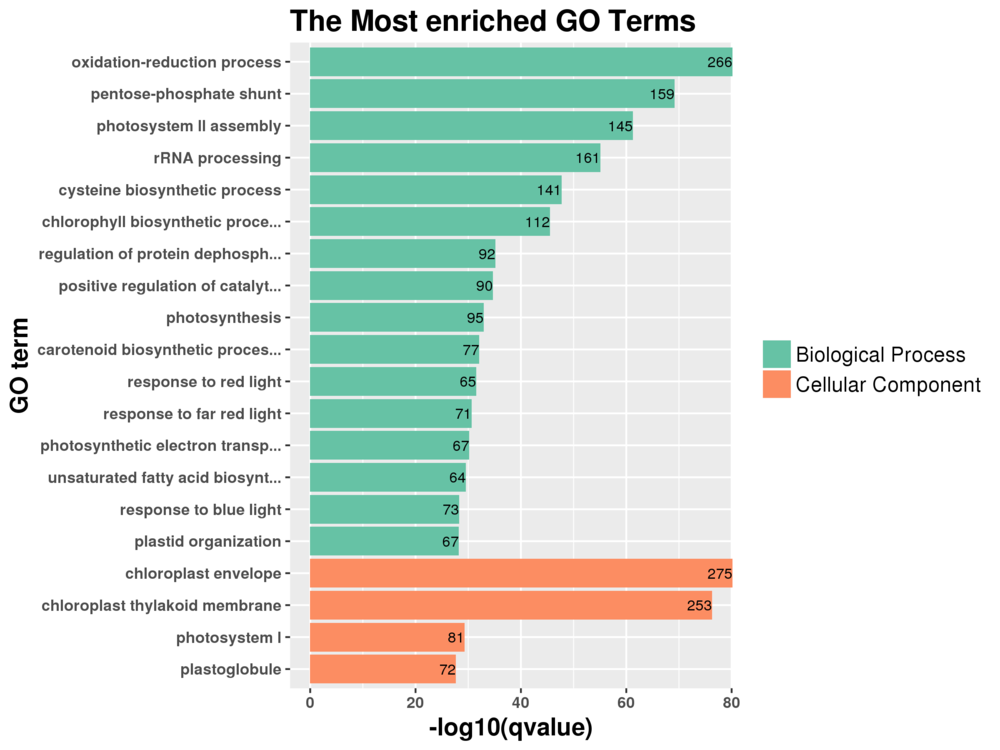

Supplement: Supplementary file 3 — Additional file 3. The Most enriched GO terms during the different time points as compared to the control. [file 12864_2020_6524_MOESM3_ESM.docx]
